# Evaluation of high-risk clinicopathological indicators in gastrointestinal stromal tumors for prognosis and imatinib treatment outcome

**DOI:** 10.1186/1471-230X-14-105

**Published:** 2014-06-07

**Authors:** Wen-Yi Zhao, Jia Xu, Ming Wang, Zi-Zhen Zhang, Lin Tu, Chao-Jie Wang, Hui Cao, Zhi-Gang Zhang

**Affiliations:** 1 Department of General Surgery, Ren Ji Hospital, School of Medicine, Shanghai Jiao Tong University, 1630 Dongfang Road, Shanghai, People's Republic of China; 2 State Key Laboratory of Oncogenes and Related Genes, Shanghai Cancer Institute, Ren Ji Hospital, School of Medicine, Shanghai Jiao Tong University, Wenxuan Building of Medicine, 800 Dongchuan Road, Shanghai, People's Republic of China

**Keywords:** Gastrointestinal stromal tumor, Mitosis, Serosal invasion, Prognosis

## Abstract

**Background:**

Although the clinical benefit of imatinib adjuvant therapy for high-risk patients with gastrointestinal stromal tumor (GIST) has been proven, the recurrence rate still remains high. This study aimed to sub-divide high-risk GIST patients with some “very high-risk” factors for more precise prognostic indicator, and possible association with efficiency of imatinib adjuvant therapy.

**Methods:**

Clinicopathological data were confirmed by pathological diagnosis and clinical records. Recurrence-free survivals (RFS) were evaluated in 370 GIST patients (212 cases as test cohort and 158 cases as validation cohort) and 48 high-risk GISTs with imatinib adjuvant therapy after R0 resection.

**Results:**

Mitosis count > 10/50 high-power fields (HPF) and serosal invasion are independent prognostic factors for RFS of GIST patients. Mitosis count > 10/50HPF and serosal invasion can sub-divide high-risk GIST patients effectively and significantly improve the area under the curve (AUC) of receiver operating characteristics (ROC) curve for prognostic indicator both in test and validation cohort. Patients with serosal invasion after R0 resection showed a poorer prognosis with imatinib adjuvant therapy.

**Conclusions:**

Sub-division of high-risk GIST patients helps to more precisely predicting the prognosis. Serosal invasion may be an adverse predictive factor in high-risk patients and imatinib treatment outcome.

## Background

Gastrointestinal stromal tumor (GIST) accounts for more than 80% of all gastrointestinal mesenchymal tumors [1]. As it ranks below only gastric and colorectal cancers, GIST is among the most common types of gastrointestinal tumors. Recently, the incidence of GIST has gradually increased [2-4] and there are more than 5000 newly diagnosed cases each year since 2011 in the United States [5].

Modified NIH criteria based on NIH consensus criteria is wildly accepted as risk-stratification scheme for GIST and four categories from very low to high risk are used to predict prognosis of GIST patients. The mitosis count, tumor size, tumor site and tumor rupture are important prognostic predictors in this scheme [6,7]. However, the clinical behaviors and the outcomes of GIST still vary even in the same group of the risk, especially in the patients with high-risk of recurrence. With wide application of imatinib mesylate (IM) in clinical practice for GIST, the mortality rate of GIST patients has decreased significantly [8]. Nevertheless, the recurrence and metastasis rates, especially for the patients at high-risk stage, remain high [8-10].

Because unclear biological behaviors and high recurrence rates in high-risk GIST patients, some of them suffer worse prognosis than others even they are classified into the same category with the same treatment. To more precisely predict prognosis and possible association with efficiency of imatinib adjuvant therapy for high-risk GIST patients, we tried to sub-divide high-risk GIST patients with some “very high-risk” factors, such as primary sites not from stomach, tumor size > 10 cm, mitosis count > 10/50HPF and serosal invasion, a common pathological diagnosis in malignant tumors. Related recurrence-free survivals (RFS) were analyzed in test and validation cohort.

## Methods

### Ethics statement

This project was approved by ethics committee of Ren Ji Hospital, Shanghai Jiao Tong University School of Medicine for the use of samples, approval No. 2012031. Informed consents were obtained from all patients before study inclusion.

### Patients and procedures

The patient inclusion criteria were as follows: 1) a distinct pathologic diagnosis of GIST; 2) underwent R0 resection, R0 resection in our study defined as margin-free resection and no metastasis detected before and during the surgery; 3) no radiotherapy, chemotherapy, nor other anti-cancer therapies prior to the surgery; and 4) availability of complete clinicopathologic and follow-up data. The patient exclusion criteria were as follows: 1) underwent R1/2 (margin-positive) resection; and 2) locally advanced GIST. The parameters, including patient age, gender, tumor site, tumor size, number of mitoses/50 high-power fields (HPF) and serosal invasion, were recorded in the official pathology database. Cases with tumor rupture were not enrolled in our study because insufficient data in our clinical records. The risk of aggressive tumor behavior was calculated according to the modified NIH criteria, which classified GIST into very low, low, intermediate, and high-risk categories.

In patients without imatinib adjuvant therapy and met the criteria in our study, were divided into two cohorts by different surgical time periods for getting more reliable results as a single center research. As the test cohort, 212 cases of GISTs (male 114 and female 98 cases; mean age: 61 years) were collected retrospectively from patients who underwent surgeries at Ren Ji Hospital, Shanghai Jiao Tong University School of Medicine from January 2010 to May 2013. In parallel, we retrospectively assessed another validation cohort collected 158 cases (male 85 and female 73 cases; mean age: 59 years) from the same hospital between January 2004 and December 2009. More details of clinic-pathological characteristics of test and validation cohort could refer to Table 1.

**Table 1:** Characteristics of GIST patients in test and validation cohort without imatinib adjuvant therapy

	**Test cohort (n = 212)**	**Validation cohort (n = 158)**	***P *** **value**
**Age (years)**			
≤ 50	36 (17.0%)	37 (23.4%)	0.124
> 50	176 (83.0%)	121 (76.6%)	
**Gender**			
Male	114 (53.8%)	85 (53.8%)	0.996
Female	98 (46.2%)	73 (46.2%)	
**Tumor site**			
Stomach	129 (60.8%)	82 (51.9%)	0.094
Small bowel	48 (22.6%)	54 (34.2%)	
Colon	10 (4.7%)	8 (5.1%)	
Others	25 (11.9%)	14 (8.9%)	
**Tumor size (cm)**			
≤ 2.0	20 (9.4%)	16 (10.1%)	0.408
2.1-5.0	100 (47.2%)	61 (38.6%)	
5.1-10.0	59 (27.8%)	54 (34.2%)	
> 10.0	33 (15.6%)	27 (17.1%)	
**Mitoses per 50 HPFs**			
≤ 5	175 (82.5%)	118 (74.7%)	0.078
6-10	23 (10.8%)	19 (12.0%)	
> 10	14 (6.7%)	21 (13.3%)	
**Modified NIH criteria**			
Very low risk	18 (8.5%)	14 (8.9%)	0.087
Low risk	96 (45.3%)	56 (35.4%)	
Intermediate risk	38 (17.9%)	24 (15.2%)	
High risk	60 (28.3%)	64 (40.5%)	
**Serosal invasion**			
Yes	23 (10.8%)	20 (12.7%)	0.591
No	189 (89.2%)	138 (87.3%)	

Complete follow-up data until December, 2013, for patients in test and validation cohort were available. RFS was calculated from the date of tumor resection until the detection of tumor recurrence or last observation. The median follow-up of the test cohort was 30 months (range, 7–49 months). In the validation cohort, the median follow-up was 69 months (range, 12–106 months). Computed tomography (CT) and/or magnetic resonance imaging (MRI) were used to verify tumor recurrence in suspected cases.

Patients with serosal invasion in our study were confirmed by the pathological diagnosis as GIST invading the layer of serosa, including the serosa from the surface of adjacent organs or tissues. An extended local excision around serosal invasion area was conducted for R0 resection. The locally advanced GIST explicitly indicated by CT or MRI before the surgery was excluded in our study because this kind of GISTs not only penetrate serosa layer but also invade parenchyma of adjacent organ always leading to R1/2 resection with very poor prognosis. Neoadjuvent imatinib therapy has already been recommended in this kind of patients by current ESMO and NCCN guidelines. Our study aimed to figure out the prognostic value of GIST which just only invading serosal layer, so local advanced GIST were excluded in our study.

The criterion of imatinib adjuvant therapy after R0 resection in our study required at least 12 months uninterrupted drugs taking with 400 mg/day. 48 high-risk cases met the criteria of imatinib adjuvant therapy since 2008 in our study. The follow-up median of the patients with imatinib therapy was 38 months (range, 16 - 71 months); *KIT* and *PDGFR* gene analysis showed 45 cases with *KIT* exon 11 and 3 cases with exon 9 mutation, and without any *PDGFR* gene mutation detected.

### Statistical analysis

Statistical analyses were conducted using SPSS for Windows (version 17.0) and MedCalc (version 11.4.2.0). For comparisons, one-way analyses of variance and chi-squared tests were performed when appropriate. RFS was calculated according to the Kaplan-Meier method. The log-rank test was used to compare the survival distributions. Univariate and multivariate analyses were based on the Cox proportional hazards regression model. Only variables that were significantly different in univariate analysis were entered into the next multivariate analysis. Receiver operating characteristics (ROC) curves were constructed to assess sensitivity, specificity, and respective areas under the curves (AUCs) with 95% CI. All statistical tests were 2-sided. *P*-value differences <0.05 were considered statistically significant.

## Results

### Mitosis count > 10/50HPF and serosal invasion are independent prognostic factors for recurrence-free survival of GIST patients

**Figure 1:**
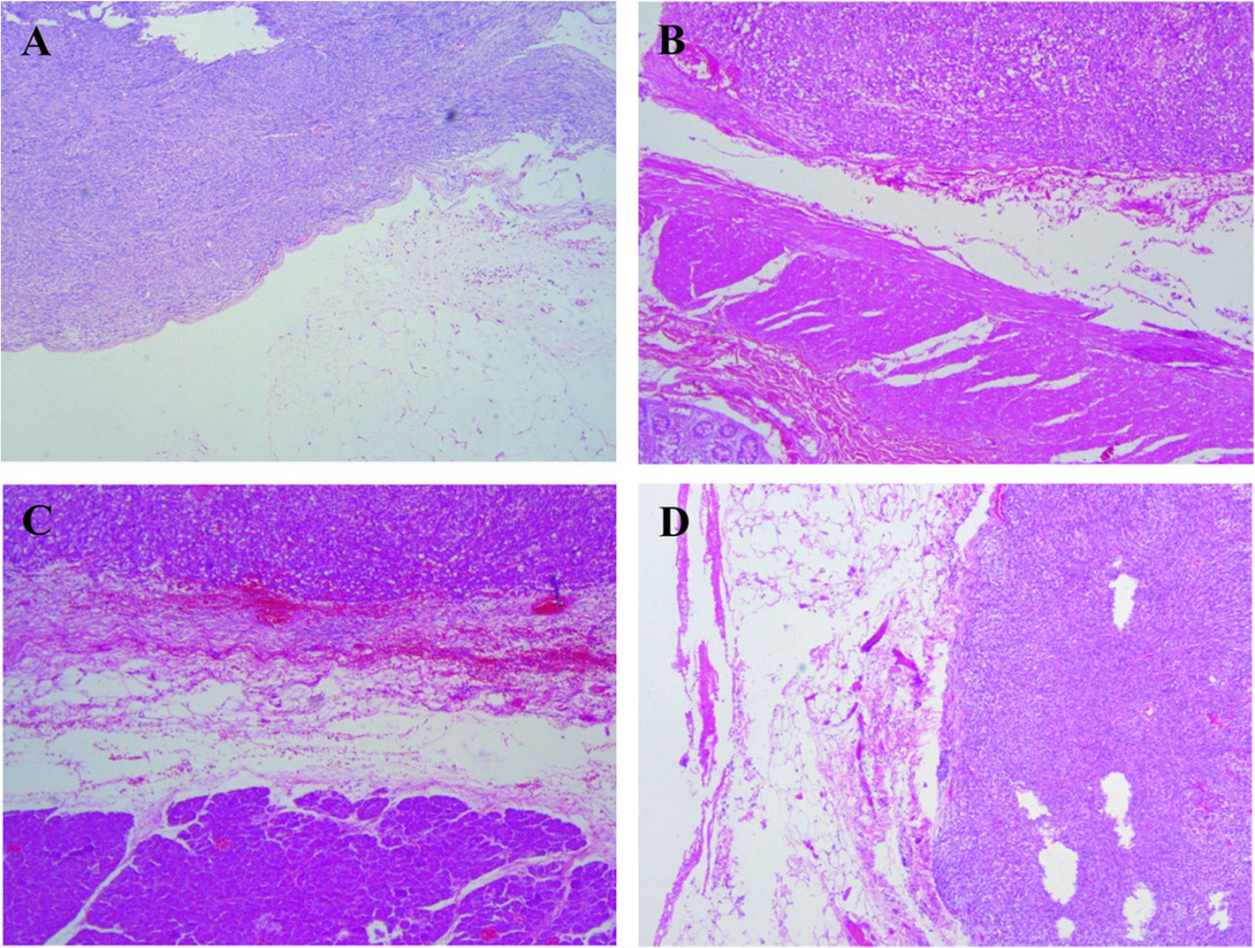
**Pathological diagnoses of serosal invasion in GISTs by Hematoxylin-Eosin staining. (A)** Intestinal GIST invading the primary serosa layer. **(B)** Intestinal GIST invading the serosa of adjacent colon. **(C)** Gastric GIST invading the pancreatic capsule. **(D)** Intestinal GIST invading the peritoneum.

Characteristics of GIST patients in test and validation cohort were shown in Table 1, chi-squared tests showed there were no differences between the test and validation cohorts in reported variables. The pathological diagnoses of serosal invasion in GISTs by Hematoxylin-Eosin staining were shown in Figure 1. Univariate analysis showed that tumor size (≤10, > 10 cm), mitosis count (≤10, > 10/50 HPF) and serosal invasion were prognostic predictors for RFS both in the test and validation cohort. Tumor site (stomach or no stomach) was identified as a prognostic predictor only in the test cohort, but not in the validation (Table 2). Furthermore, the multivariate analysis found that mitosis count > 10/50 HPF and serosal invasion were independently unfavorable prognostic factors for RFS both in test and validation cohort (Table 3).

**Table 2:** Univariate analyses of factors by cox regression model associated with recurrence-free survival (RFS) in test and validation cohort (*, P < 0.05; **, P < 0.01)

	**Test cohort**		**Validation cohort**	
**Variable**	**RFS hazard ratio (95% Cl)**	***P *** **value**	**RFS hazard ratio (95% Cl)**	***P *** **value**
Age (≤50, > 50)	0.463 (0.178-1.206)	0.115	3.673 (1.126-11.983)	0.031*
Gender (male, female)	0.594 (0.237-1.489)	0.266	0.342 (0.161-0.728)	0.005**
Tumor site (stomach, no stomach)	9.629 (2.820-32.879)	<0.001**	1.760 (0.900-3.440)	0.098
Tumor size (≤10, > 10 cm)	8.732 (3.604-21.154)	<0.001**	5.392 (2.787-10.430)	<0.001**
Mitosis count (≤10, > 10/50 HPF)	13.459 (5.470-33.115)	<0.001**	8.462 (4.335-16.519)	<0.001**
Serosal invasion (Yes, No)	20.531 (8.170-51.596)	<0.001**	7.706 (3.938-15.081)	<0.001**

**Table 3:** Multivariate analyses of factors by cox regression model associated with recurrence-free survival (RFS) in test and validation cohort (*, P < 0.05; **, P < 0.01)

	**Test cohort**		**Validation cohort**	
**Variable**	**RFS hazard ratio (95% Cl)**	***P *** **value**	**RFS hazard ratio (95% Cl)**	***P *** **value**
Tumor size (≤10, > 10 cm)	1.525 (0.465-5.001)	0.486	2.084 (0.901-4.821)	0.086
Mitosis count (≤10, > 10/50 HPF)	3.388 (1.193-9.636)	0.022*	4.117 (1.845-9.185)	0.001**
Serosal invasion (Yes, No)	10.220 (2.862-36.493)	<0.001**	2.960 (1.237-7.079)	0.015*

### Mitosis count > 10/50HPF and serosal invasion can sub-divide high-risk GIST patients effectively and significantly to improve the prognostic indicator

**Figure 2:**
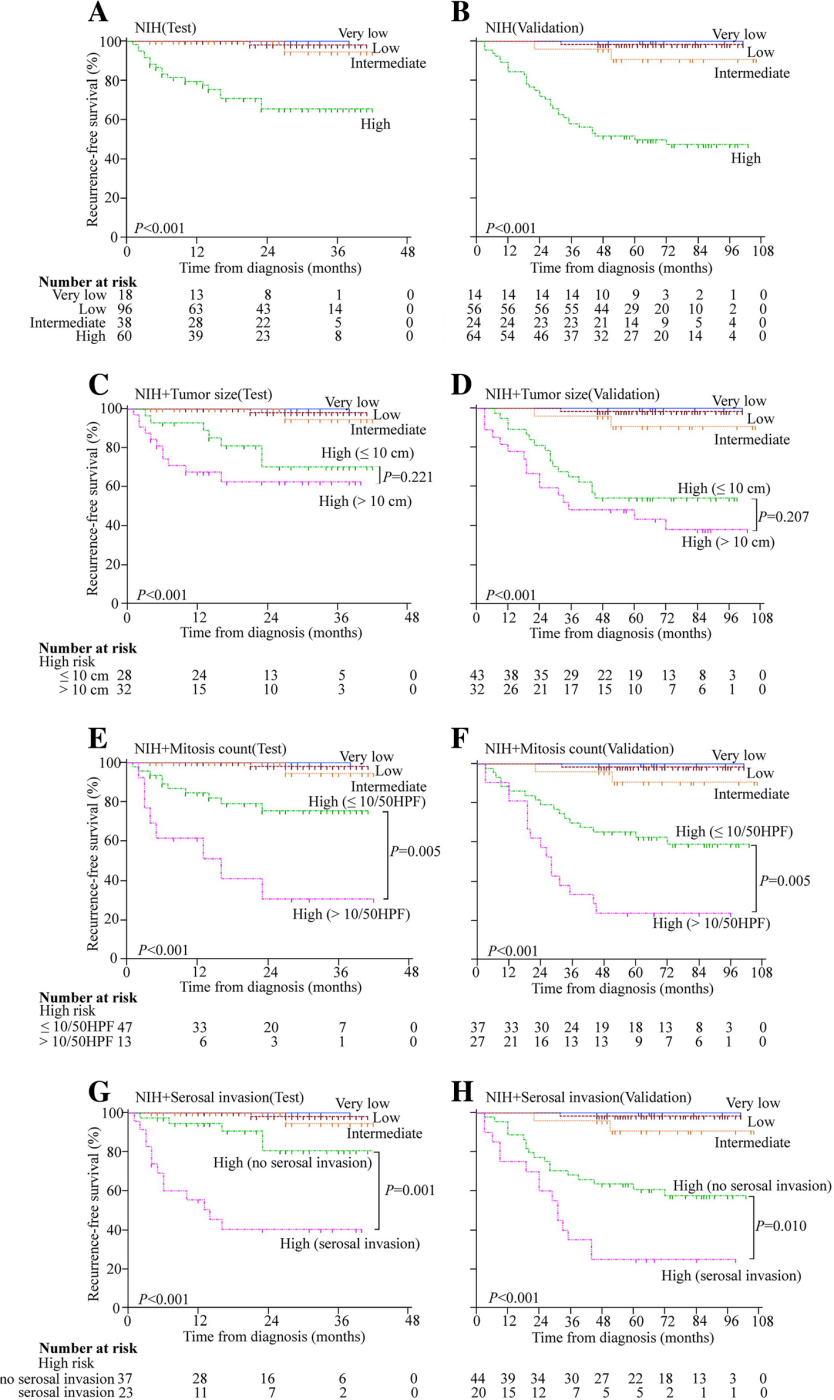
Prognostic significance by modified NIH criteria in test (A) and validation (B) cohort, tumor size sub-groups in test (C) and validation (D) cohort, mitosis count sub-groups in test (E) and validation (F) cohort and serosal invasion sub-groups in test (G) and validation (H) cohort in GISTs.

Since the aforementioned factors were prognostic predictors or independently unfavorable prognostic factors for RFS of GIST patients, the further study was focused on whether these factors can sub-divide high-risk GIST patients and improve the AUC of the ROC curve for prognostic indicator. To test our hypothesis, the high-risk GIST patients were divided into the following sub-groups according to tumor size (≤10 cm vs > 10 cm subgroup), mitosis count (≤10 HPF vs > 10/50 HPF subgroup) and serosal invasion (serosal invasion *vs* no serosal invasion subgroup). RFS of GIST patients in test and validation cohort classified by modified NIH criteria were shown in Figure 2A and B. Log-rank test showed that tumor size (≤10 or > 10 cm) can not sub-divide high-risk group effectively both in test and validation group (*P* > 0.05) (Figure 2C and D). Mitosis count > 10/50 HPF and serosal invasion can significantly sub-divide high-risk group as unfavorable prognostic factors both in test and validation group (*P* < 0.05) (Figure 2E, F, G and H).

**Figure 3:**
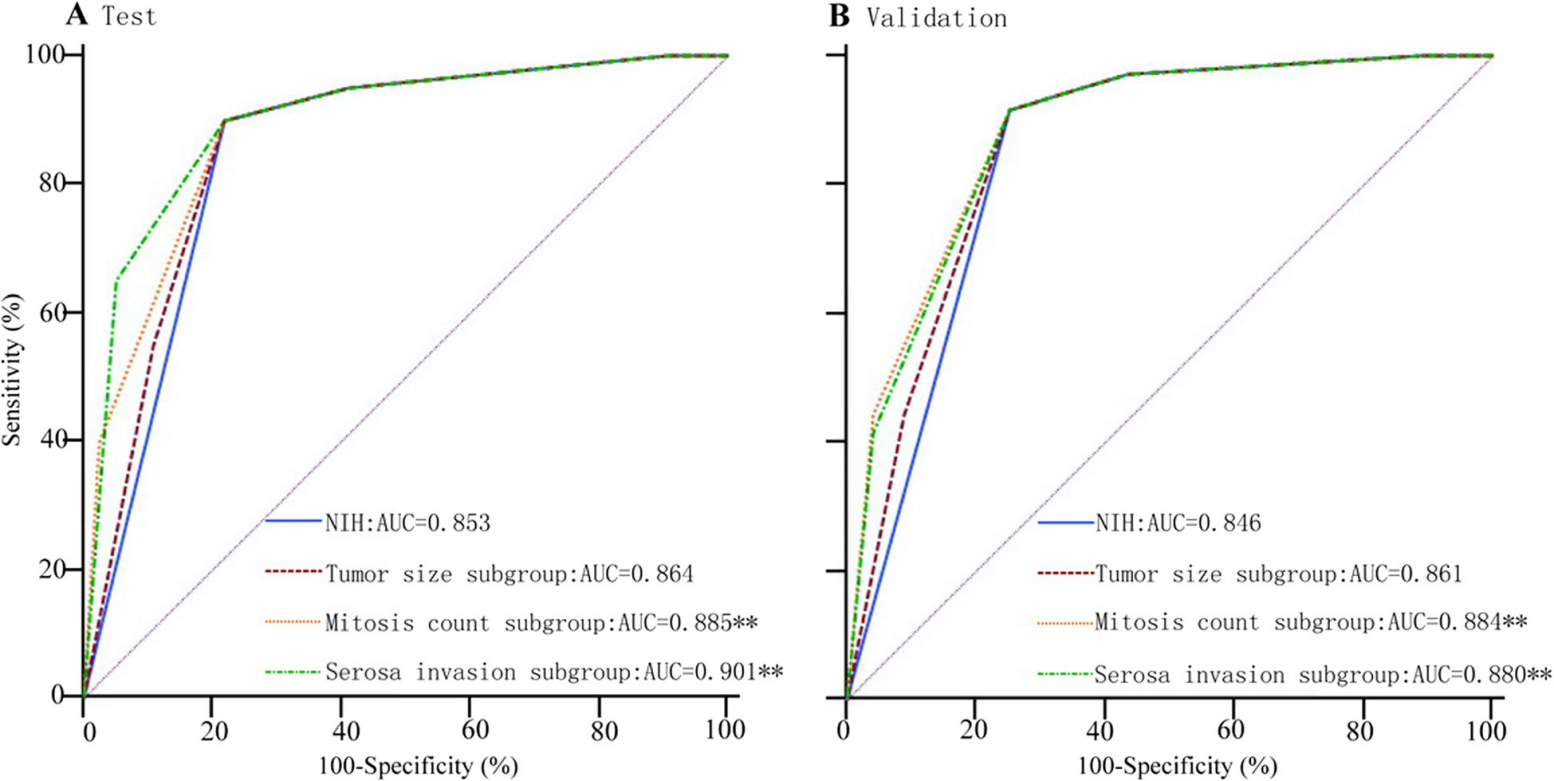
ROC curve for modified NIH criteria versus tumor size sub-group, mitosis count sub-group and serosal invasion sub-group in prognostic indicator in test (A) and validation (B) cohort. (**, *P* **< 0.01).**

ROC analysis showed that subdivided mitosis count or serosal invasion based on modified NIH criteria can improve modified NIH criteria for prognostic indicator in GIST. AUC of subdivided mitosis count (0.885 for test cohort and 0.884 for validation cohort) were higher than that of modified NIH criteria (0.853 for test cohort and 0.846 for validation cohort) with significantly difference both in the test and the validation cohort (*P* < 0.01). The AUC of serosal invasion (0.901 for test cohort and 0.880 for validation cohort) were also significantly higher in both the test and the validation cohort (*P* < 0.01) (Figure 3).

### Serosal invasion may act as a predictive factor for the efficacy of imatinib adjuvant therapy

**Figure 4:**
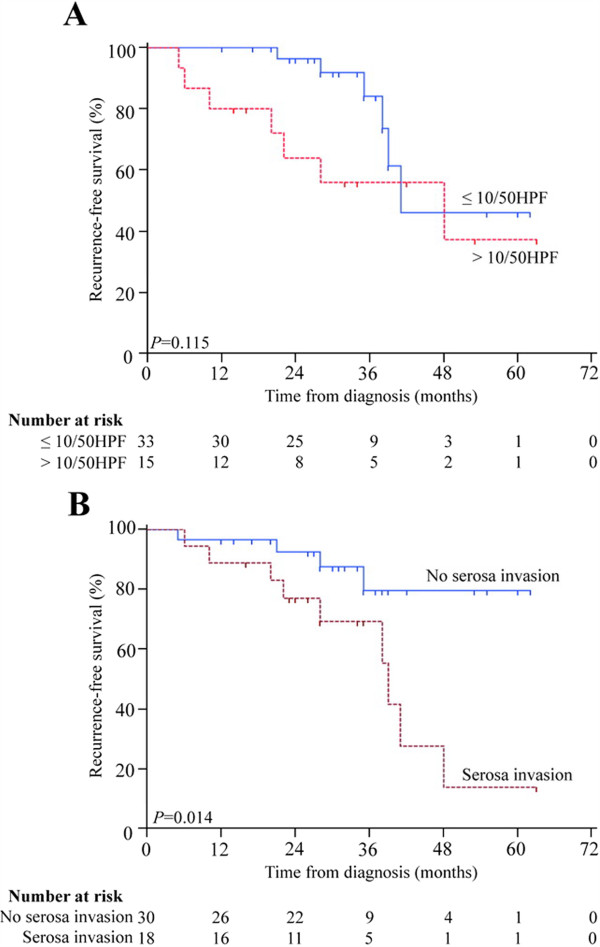
**Recurrence-free survival (RFS) in high-risk patients with imatinib adjuvant therapy. (A)** RFS by mitosis count > 10/50 HPF in patients with imatinib adjuvant therapy. **(B)** RFS by serosal invasion in patients with imatinib adjuvant therapy.

Since high-risk GIST patients after R0 resection indicated adjuvant therapy of imatinib, we further investigated whether mitosis count > 10/50 HPF or serosal invasion could affect the efficacy of imatinib adjuvant therapy. Kaplan-Meier survival analysis with log-rank test showed there was no difference in RFS between mitosis count > 10/50 HPF nor ≤ 10/50 HPF subgroup with imatinib therapy (*P* = 0.115). But the RFS of patients with serosal invasion showed sharp decline of the curve as opposed to those without serosal invasion, indicating serosal invasion as unfavorable effect on imatinib adjuvant therapy (*P* = 0.014) (Figure 4). The multivariate analysis found that serosal invasion was independently unfavorable prognostic factors for RFS of the GIST patients with imatinib adjuvant therapy (Table 4).

**Table 4:** Multivariate analyses of factors associated with recurrence-free survival (RFS) in GIST patients with adjuvant imatinib therapy (*, P < 0.05)

**Variable**	**RFS hazard ratio (95% Cl)**	***P *** **value**
Tumor size (≤10, > 10 cm)	0.801 (0.222-2.889)	0.736
Mitosis count (≤10, > 10/50 HPF)	1.944 (0.546-6.913)	0.307
Serosal invasion (Yes, No)	3.549 (1.047-12.025)	0.043*

## Discussion

GIST has a wide various biological behaviors with malignant potential, so it can not be precisely distinguished as benign or malignant lesions. Mitotic index, tumor size, tumor site and tumor rupture which are from modified NIH criteria can be important prognostic predictors of GISTs [6,7,11]. According to the NIH guidelines, all GISTs might have malignant potential. Moreover the recurrence risk in high-risk cases was significantly higher than that in intermediate-, low- and very low-risk cases, and even in the same high-risk classification, the clinical outcomes of GIST patients are always variety in our approximate 10 years’ follow-up database [12,13]. For example, patients with GIST invading local tissue or organ even if receiving R0 resection always appear poor prognosis. On the contrary, some cases with large tumor size but less mitosis count and tumor well-encapsulated are no sign of recurrence for a long time follow up (>5 years) without imatinib adjuvant therapy. As we all know, there is no perfect criteria which can predict the prognosis of the disease with 100% accuracy, and there must be some unrevealed room for improving or complementing the current criteria.

The aim of this study is to find “very high-risk” factors which can effectively sub-divide high-risk patients after R0 resection for more precisely predicting prognosis. Because our purpose was for clinical application, the factors we chose must be easily understandable, well-accepted and feasible variables. Primary sites not from stomach, tumor size > 10 cm and mitosis count > 10/50HPF were selected by referring to modified NIH criteria [7], serosal invasion was also selected in our study as a common pathological diagnosis in malignant tumors.

Tumor rupture is a poor prognostic factor indicated by modified NIH criteria. Tumor rupture are divided into two clinical conditions, one is spontaneous tumor rupture prior to operation, and another is a result of the manipulation at surgery [14,15]. Tumor rupture was not enrolled in our study because insufficient data in our clinical records. This situation was common in other centers as in Joensuu et al.’s multi-center research in which data of tumor rupture were unavailable from 53.2% cases in pooled population-based cohort and 100% cases in validation [11]. Actually most patients with spontaneous tumor rupture before surgery frequently found having already accompanied with miliary nodules as implantation metastasis and impossible for R0 resection were excluded in our study. Another condition, if iatrogenic tumor rupture, mainly depends on surgeon’s subjective judgment and active reporting. Serosal invasion is a common pathological diagnosis in malignant tumors but few mentioned in GIST before [16], the merits of serosal invasion we selected in this study were less interference with subjective factors and surgical matters compared with tumor rupture, easily observed in surgery and feasibly confirmed by pathological diagnosis. But considering serosal invasion is not a standard prognostic factor in GIST enrolled in the guidelines yet, it mostly depends on detection and reporting by pathologists. Mitosis count is also a strong prognostic factor in GIST and enrolled in NIH criteria, but it still has limitations and its reliability is controversial. Identification of mitoses should be subjective, and the number detected depends on the tissue fixation time and the magnification of the field under the microscope [11].

Through univariate and multivariate analyses of the factors associated with RFS in test and validation cohort with GIST, mitosis count > 10/50HPF and serosal invasion were confirmed as the independent prognostic factors for the RFS. The Kaplan-Meier survival analysis with log-rank test showed sub-dividing high-risk GIST patients by mitosis count > 10/50HPF and serosal invasion yielded significantly different outcomes and more effectively differentiated the groups of GIST patients.

The prognostic accuracy of modified NIH criteria and sub-groups based on modified NIH criteria were compared using ROC analyses, mitosis count or serosal invasion sub-group, produced better estimats for the risk of GIST recurrence. The AUC was larger for the mitosis count (0.890, 95% CI 0.841-0.968) or serosal invasion sub-groups (0.905, 95% CI 0.858-0.940) than that for modified NIH criteria in test cohort (0.860, 95% CI 0.807-0.902; *P* < 0.01), and the results remained similar in the validation. Criteria of prognostic indicator is always required the accuracy as high as possible but the schemes as simple as possible to meet various clinical cases. Our study demonstrated two common factors, mitosis count > 10/50HPF and serosal invasion. One of them already has been included in high risk schemes of NIH criteria.

We further focused on whether mitosis count > 10/50HPF or serosal invasion could affect efficiency of imatinib adjuvant therapy. The results showed that GIST patients with serosal invasion might suffer a poorer prognosis with imatinib therapy, but there was no difference between patients with mitosis count > 10 and mitosis count ≤ 10/50HPF in the RFS. Because of the limitation of the sample numbers and follow-up time in our study, it still needs more works to verify this result, but the high-risk GIST patients with serosal invasion should be noticed in clinical follow-up because of higher possibility in recurrence even with imatinib adjuvant therapy.

How to improve outcomes of GIST patients with serosal invasion even after R0 resection is still a serious problem. Should this kind of patients need more than 3-year imatinib therapy? The real benefits and optimal time to stop treatment still need further study and it still remains the possibility of recurrence after stopping taking the drugs. Neoadjuvant imatinib therapy, which has been recommended by current ESMO and NCCN guidelines for locally advanced GIST [17,18], may help to improve the outcomes of GIST patients with serosal invasion. Neoadjuvant imatinib therapy can shrink the size of locally advanced GISTs and increase R0 resection rate, which significantly prolong the RFS and OS comparing with the inoperable patients or patients with palliative operation [19-23]. But there are still some problems that need to be solved in future study: first, how to verify serosal invasion of GISTs before surgery? Serosal or subserosal invasion are as superficial infiltration different from typical locally advanced GIST which can be judged by CT or MRI easily. It is hard for imaging examination to distinguish the real invasion or tumor just leaning against adjacent organs or tissues; for effective neoadjuvant therapy, aspiration biopsy is necessary for not only diagnosis but also gene mutation detection for *KIT* and *PDGFR*, but conducting biopsies in such highly malignant GIST may increase the risks of tumor rupture or implantation metastasis.

## Conclusions

In this study, we analyzed several “very high-risk” factors in high-risk group of GIST patients after R0 resection. Serosal invasion appears to improve the modified NIH criteria and predicts an unfavorable outcome in high-risk GIST patients, with or without imatinib treatment. Our study demonstrated the possibility of personalized medical strategy for different GISTs by sub-dividing high-risk patients.

## Abbreviations

GIST: Gastrointestinal stromal tumor; IM: Imatinib mesylate; HPF: High-power fields; RFS: Recurrence-free survival; CT: Computed tomography; MRI: Magnetic resonance imaging; ROC: Receiver operating characteristics; AUCs: Respective areas under the curves.

## Competing interests

The authors declare that they have no competing interests.

## Authors’ contributions

W-YZ, HC, Z-GZ made the conception, design and drafted the manuscript; W-YZ, JX, MW, Z-ZZ, LT and C-JW carried out collection and assembly of data; All authors read and approved the final manuscript.

## Pre-publication history

The pre-publication history for this paper can be accessed here:

http://www.biomedcentral.com/1471-230X/14/105/prepub
